# Security and Privacy Qualities of Medical Devices: An Analysis of FDA Postmarket Surveillance

**DOI:** 10.1371/journal.pone.0040200

**Published:** 2012-07-19

**Authors:** Daniel B. Kramer, Matthew Baker, Benjamin Ransford, Andres Molina-Markham, Quinn Stewart, Kevin Fu, Matthew R. Reynolds

**Affiliations:** 1 Department of Medicine, Beth Israel Deaconess Medical Center, Harvard Medical School, Boston, Massachusetts, United States of America; 2 Department of Computer Science, University of Massachusetts, Amherst, Massachusetts, United States of America; Los Angeles Biomedical Research Institute, United States of America

## Abstract

**Background:**

Medical devices increasingly depend on computing functions such as wireless communication and Internet connectivity for software-based control of therapies and network-based transmission of patients’ stored medical information. These computing capabilities introduce security and privacy risks, yet little is known about the prevalence of such risks within the clinical setting.

**Methods:**

We used three comprehensive, publicly available databases maintained by the Food and Drug Administration (FDA) to evaluate recalls and adverse events related to security and privacy risks of medical devices.

**Results:**

Review of weekly enforcement reports identified 1,845 recalls; 605 (32.8%) of these included computers, 35 (1.9%) stored patient data, and 31 (1.7%) were capable of wireless communication. Searches of databases specific to recalls and adverse events identified only one event with a specific connection to security or privacy. Software-related recalls were relatively common, and most (81.8%) mentioned the possibility of upgrades, though only half of these provided specific instructions for the update mechanism.

**Conclusions:**

Our review of recalls and adverse events from federal government databases reveals sharp inconsistencies with databases at individual providers with respect to security and privacy risks. Recalls related to software may increase security risks because of unprotected update and correction mechanisms. To detect signals of security and privacy problems that adversely affect public health, federal postmarket surveillance strategies should rethink how to effectively and efficiently collect data on security and privacy problems in devices that increasingly depend on computing systems susceptible to malware.

## Introduction

Medical devices play a growing role in the care of millions of patients worldwide.[1], [2] Devices for diseases ranging from heart failure to diabetes improve patient outcomes and may ease disease management.[3] Recent innovations in medical device design include more complex diagnostics and the storage of patient data. In many cases, this information can be transmitted directly to physicians or indirectly through a third-party provider, sometimes using wireless systems, to assist with diagnosis and management of chronic medical problems. At present, information flow between implanted devices and providers is predominantly unidirectional (from device to provider). Theoretically, however, current technologies could easily be modified such that remote interactions between providers and medical devices (e.g. to reprogram an insulin pump or pacemaker) would be possible. The possibility of hacking into an insulin pump was recently demonstrated by a Type 1 diabetic on his own device.[4].

The rapid dissemination of medical devices capable of storing and transmitting patients’ medical information and the theoretical possibility of remotely reprogramming implanted medical devices raise important concerns regarding security, privacy, and safety.[5] Investigators have demonstrated limitations of the security functions for implantable cardioverter-defibrillators (ICDs), for example, by proving the feasibility of communicating with an ICD through an unauthorized radio-based approach that theoretically could interfere with appropriate device therapy.[6] While there are hundreds of confirmed reports of conventional computer viruses infecting medical devices in radiology, cardiac catheterization labs, sleep labs, and other clinical departments, there are no known case reports of malevolent interference that specifically target medical device function.[7], [8] A growing list of confirmed cybersecurity vulnerabilities in medical devices pose challenging risks to patients whose privacy or disease management depends on the proper functioning of devices.

In the United States, post-market surveillance of medical devices identifies potential risks and connects device malfunction to adverse events in patients. Post-market events may trigger recalls or advisories depending on the nature of the device problem that is identified.[9] These reports may provide important information about safety and effectiveness, and have led to revision of regulatory practices for devices such as ICD leads and automated external defibrillators.[10], [11].

In order to better understand the security vulnerabilities of marketed medical devices, we performed an analysis of recalls and adverse events, which we adjudicated to identify post-market actions related to security or privacy, and to identify specific features of devices at risk for recalls with security implications.

## Methods

**Figure 1:**
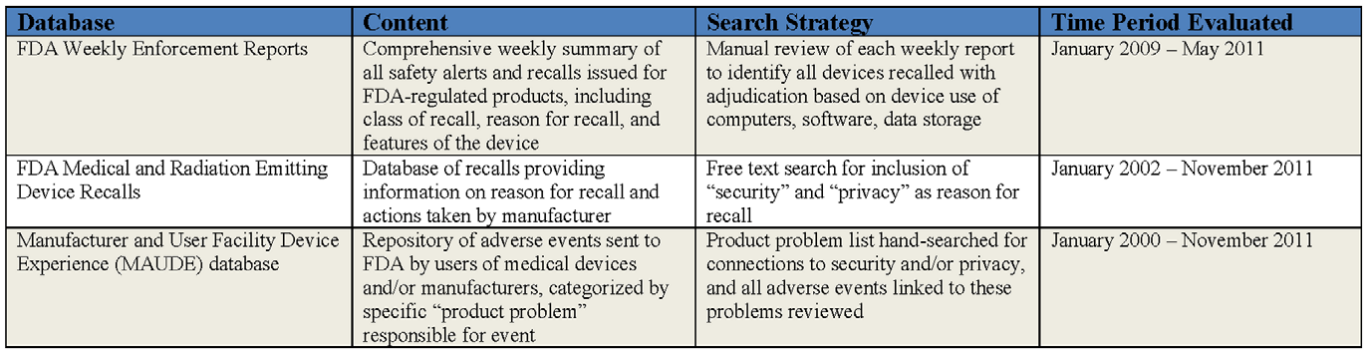
Summary of Sources for Search Strategy.

We used publicly available databases maintained by the Food and Drug Administration (FDA). **Figure 1** summarizes the different sources leveraged for our analysis.

### FDA Enforcement Reports

In order to identify a comprehensive denominator of medical device recalls, we accessed publicly available weekly enforcement report listings on the FDA website.[12] These reports are published on a weekly basis and contain information regarding actions emerging from agency regulation. Actions include Safety Alerts and Recalls. Safety alerts are communications issued by a manufacturer, distributor, or other responsible party or the FDA to inform health professionals or other appropriate persons or firms of a risk of substantial harm from a medical device in commercial use. Recalls are issued by the FDA when a reasonable likelihood of causing harm exists, and are classified according to the likelihood of causing patient harm. Class I recalls are the most serious, indicative of situations in which there is a “reasonable probability that the use of or exposure to a violative product will cause serious adverse health consequences or death.” Class II and III recalls are less serious. Enforcement reports may also include notice of civil or criminal proceedings or seizures of products.

FDA Enforcement Reports from January 2009 through May of 2011 were reviewed, and all actions other than recalls related to medical devices (such as those specific to food or drugs) were excluded. Reports were manually searched for agency actions related to medical devices, and details from each report were extracted. These features included the specific device and device type, organ system, manufacturer, date and class of recall, estimated volume of distribution, and the reason for the recall itself. We categorized these reasons as follows: sterility/contamination; mechanical failure; electrical failure; software failure; computer hardware failure; instruction or manual mislabeling; unapproved usage; incorrect shelf life; or naming problems. We also categorized each device according to specific features including: permanent implantation; inclusion of a computer; ability to communicate wirelessly; and storage of personal data. Similarly, each recall was adjudicated as to whether or not the reason for recall was related to these specific functions (e.g. personal data storage, wireless communication, etc.).

### Device Recalls

The FDA also hosts a public, searchable database of Medical and Radiation Emitting Device Recalls, which houses information related to recalls of medical devices since November 2002.[13] Information that can be extracted from this database includes the date and a narrative explanation of the reason for the recall and details of actions taken by the manufacturer. The FDA recall database can be searched by date, manufacturer, recall class or number, or the reason for recall using a free-text search window. Terms such as “battery failure”, “labeling”, “sterility”, etc. can be used to identify recalls related to specific problems or malfunctions with a device. We searched using “security” and “privacy” as search terms to identify recalls where either of these elements were considered central or important.

### Adverse Event Reports

Lastly, we used the Manufacturer and User Facility Device Experience (MAUDE) database to look for adverse events related to security or privacy problems.[14] The MAUDE database was established through the Safe Medical Devices Act of 1990, and requires sites where medical devices are used (hospitals, nursing homes, physicians’ offices, etc.) to report device-related fatalities and serious adverse events directly to FDA as well as to the manufacturers.[15] Since 1995, these reports have been stored in a searchable, publicly available database. This database is used for FDA analysis and is also available to independent researchers. The majority (>90%) of these reports come from manufacturers, with the remainder submitted by user facilities, such as hospitals and outpatient clinics or individual physicians. Manufacturers are required to report any adverse events that are communicated to them verbally or in writing. These reports include details related to the device type and model number, timing and location of adverse events, clinical details, and description of the manufacturer’s analysis of the device (if available).

MAUDE can be searched using its advanced interface, which provides a drop-down menu of “product problems” from which to choose. The advanced interface includes approximately 1000 product problem terms. These were manually searched and evaluated for inclusion of “security” and “privacy”, and each term was also evaluated individually for a plausible relationship to either theme. Any adverse events mapped to those search terms related to security or privacy were then reviewed in detail. Adverse events from January 1, 2000 through November 30, 2011 were included in the searches.

### Software Recalls Analysis

Preliminary analysis of enforcements reports identified software related recalls as a particularly prevalent problem with potential security and safety ramifications. Thus, all software-related recalls were identified using the searchable FDA recall database between 2002 and 2010 for those recalls that included the word “software” in their reason for recall. The results included Class I, II, and III recalls. For each of these software related recalls, we determined whether the recall cited problems in the software itself as the reason for the recall, as opposed to problems with labeling alone. We noted whether the recall mentioned that a software update was to be issued and whether this software update was mentioned as being available online, or explicitly mentioned as not being available online. We also recorded whether the software update, if available, involved a manufacturer representative visiting the installation site or return of a device to the manufacturer by mail.

## Results

### Enforcement Reports

We identified 1845 recalls issued from January 2009 to May 2011 from the weekly enforcement reports listings. **TABLE 1** summarizes the categorization of recalls by organ system and etiology. For all recalls of the organ systems or usages involved most commonly were laboratory/pathology (294, 15.9%), orthopedic (279, 15.1%), cardiovascular (250, 13.5%) and general hospital (225, 12.2%). The most common reasons for recalls were mechanical problems (918, 49.8%) and software problems (279, 15.1%).

**Table 1:** Characteristics of Weekly Enforcement Reports, 2009–2011 (N = 1845).

**Common organ system/usage**	Laboratory/pathology (294, 15.9%)
	Orthopedic (279,15.1%)
	Cardiovascular (250, 13.5%)
	General Hospital (225, 12.2%)
	Radiology (164, 8.9%)
	General Surgery (121, 6.6%)
**Permanent Devices**	241 (13.1%)
**Devices with computer functions**	605 (32.8%)
**Devices capable of wireless communication**	35 (1.9%)
**Storage of personal data**	31 (1.7%)
**Selected recall reasons**	Mechanical Problem (918, 49.8%)Software problems (279, 15.1%)Instruction or manual mislabeling (268, 14.5%)Sterility/contamination (185, 10.0%)Electrical failure (82, 4.4%)Computer hardware failure (17, 0.9%)

Permanent implants were the subject of 241 (13.1%) recalls. Of the recalled devices, 605 (32.8%) included computers, but only 35 (1.9%) stored patient data and 31 (1.7%) were capable of wireless communication. Though storage of patient data and wireless communication were relatively uncommon features of implanted devices, these features were often adjudicated to be responsible for recalls of devices utilizing them. For example, 301 (49.8%) of devices with computers that were recalled had computing functions as the reason for the recall itself. Six (17.1%) of the 35 devices storing patient data had recalls originating from this function, and 6 (19.4%) of the 31 devices using wireless communication had recalls originating from this function.

An example of one of the enforcement reports (from November 2010) is for a PC Unit for use with infusion and monitoring systems. The reason for the recall provided in the report is: “Under certain wireless network conditions a communication error can occur, which freezes the PC Unit screen, which may result in a delay of therapy. A delay of therapy may result in serious injury and/or death”.[16]

An example of a software related enforcement report corresponds to an ultrasound system. The reason for the recall is listed as: “The product has a software problem in which previous patient measurement data gets associated with another patient’s image”. [17]

### Recall Searches

“Security” as a search term for recalls returned only one finding. This was a Class II recall, for a radiation oncology system including a console and software to confirm proper patient positioning for therapy. The MAUDE report for this recall was incomplete, but alluded to a failure of security measures designed to restrict access to the console (incomplete sentence quoted verbatim): “The Operator Station Calibration panel provides access to view and modify machine specific configuration settings. Access to these settings has always been restricted to individuals with appropriate security rights, being limited to only the ‘Superuser’ and ‘Field Service engine’”.[17] “Privacy” did not return any reported adverse events.

### MAUDE Searches

Manually searching the advanced interface of MAUDE yielded “Computer system security problem”; “patient data issue”; and “unauthorized access to computer system” as the only terms (out of nearly 1000) that were related by title to security and privacy features of devices. Adverse events from each of these terms from January 1, 2000 through November 30, 2011 were reviewed. Importantly, despite categorization in MAUDE under these headings, review of the specific adverse events revealed that only one of them was actually related to privacy or security in even a tangential way. **TABLE 2** describes the exact contents of each MAUDE entry, including the device type and manufacturer narrative of the device problem. These are of variable detail (see **TABLE 2**), and range from no data at all (as with the “powered wheelchair” entry included under “computer system security problem” and specifics of an esophageal implant problem categorized as “patient data issue”. “Computer system security problem” yielded 4 reports, none of which on review was related to either computers or security. “Patient data issue” yielded 5 reports, only one of which clearly had security and/or privacy implications. In the one pertinent “patient data issue” case, a remote monitoring system for an implantable cardioverter-defibrillator routed patient information to a physician practice from which the patient no longer received follow-up care. “Unauthorized access to computer system” did not yield any reports.

**Table 2:** Adverse Event Reports from MAUDE Linked to Security or Privacy Problems.

Product Problem category	Event Date	Device Type	Device/Manufacturer	Verbatim Text	Adjudicated Security or Privacy Implications?
**“Computer system security issue”**					
	9/29/2011	Powered wheelchair	**INVACARE TAYLOR STREET POWERED WHEELCHAIR 890.3860**	None	No
	4/8/2010	Orthopedic implant	**DEPUY ORTHOPAEDICS, INC. ENDURON NEUT 54OD X 28ID**	**Manufacturer Narrative**	No
				This complaint is still under investigation. Depuy will notify the fda of the results of this investigation once it has been completed.	
				**Event Description**	
				Enduron liner has failed. Excessive wear causing extensive osteolysis.	
	2/9/2010	Orthopedic implant	**DEPUY ORTHOPAEDICS, INC. AMK PATELLA 8.5 X 34MM 87 JWH**	**Manufacturer Narrative**	No
				This complaint is still under investigation. Depuy will notify the fda of the results of this investigation once it has been completed.	
				**Event Description**	
				Pt was revised to address femoral and tibial loosening. Poly wear and osteolysis were discovered intraoperatively.	
	1/11/2010	Orthopedic implant	**DEPUY ORTHOPAEDICS, INC. UNKNOWN DEPUY DURALOC LINER TOTAL HIP REPLACEMENT**	The devices associated with this report were not returned. Review of the device history records and/or a complaint database search was not possible as the product and lot codes required were unavailable. The investigation could not draw any conclusions regarding the reported event with the info available. Based on the investigation, the need for corrective action is not indicated. Depuy considers the investigation closed at this time. Should the product and/or additional information be received to change the outcome of the performed investigation, the complaint will be re-opened.	No
				**Event Description**	
				Patient was revised to address femoral stem loosening. Poly wear and osteolysis were discovered intraoperatively.	
**“Patient data issue”**					
	6/3/2011	Cardiac device monitoring system	**MEDTRONIC, INC. PACEART SYSTEM SOFTWARE**	**Manufacturer Narrative**	Yes
				The information submitted reflects all relevant data received. If additional relevant information is received, a supplemental report will be submitted.	
				**Event Description**	
				It was reported that a carelink patient followed at another practice in a different state had a transmission continue to pull into this practice’s paceart data exchange log viewer. The paceart issue was resolved. No patient complications have been reported as a result of this event.	
	3/4/2011	Cardiac device monitoring system	**MEDTRONIC, INC. PACEART SYSTEM SOFTWARE**	**Event Description**	No
				It was reported that a remote transmission of a patient’s device had discrepancies with the remote event in the electronic medical records system. No patient complications have been reported as a result of this event.	
				**Manufacturer Narrative**	
				The information submitted reflects all relevant data received. If additional relevant information is received, a supplemental report will be submitted.	
	5/13/11	Esophageal stent	**BOSTON SCIENTIFIC - GALWAY ULTRAFLEX ESOPHAGEAL NG STENT SYSTEM PROSTHESIS, ESOPHAGEAL**	**Manufacturer Narrative**	No
				Although the exact patient age is unknown, the patient was reported to be over 18 years of age. The complainant indicated that the device was implanted and will not be returned for evaluation; therefore, a failure analysis of the complaint device cannot be completed. If any further relevant information is identified, a supplemental medwatch will be filed.	
				**Event Description**	
				It was reported to boston scientific corporation that an ultraflex esophageal covered stent was implanted during an esophageal stenting procedure on (b)(6), 2011. According to the complainant, the indication for the stent placement was esophageal cancer. The label on the packaging of the stent stated that the stent was 7 cm in length and covered. However, following the stent placement, the user believed the stent to be uncovered. The stent position was adjusted with rat-tooth forceps and the stent was left implanted. There were no patient complications as a result of this event. The patient condition at the conclusion of the procedure was reported to be stable. Attempts to obtain additional information regarding the circumstances surrounding this event have been unsuccessful to date. Should additional relevant details become available, a supplemental report will be submitted.	
	11/9/2010	Pulmonary function test calculator	**HOSPIRA POINT OF CARE SOLUTIONS ENDO TOOD SOFTWARE**	None	No
	9/3/2010	Automated white blood cell differential counter	**ABBOTT DIAGNOSTICS DIVISION CELL-DYN SAPPHIRE ANALYZER AUTOMATED HEMATOLOGY ANALYZER**	**Event Description**	No
				The customer observed that occasionally, barcoded patient samples processed using a cell-dyn sapphire analyzer would be incorrectly mismatched to the specimen id number and wrong patient name. Sample (b)(6) was replicated by the cd sapphire and potentially mismatched to an incorrect patient name. The customer uses a laboratory information system (lis) to further process patient data. No mismatched results or incorrect reports were released from the lab. No adverse patient outcomes were reported related to this issue.	
				**Manufacturer Narrative**	
				(b)(4). An investigation is in process. A follow-up report will be submitted when the investigation is complete.	

### Software Related Recalls

From 2002 through 2010, 523 of the 537 recalls (97.4%) that mentioned the word “software” cited software specifically as the reason for the recall. Of these, 428 (81.8%) mentioned a software upgrade, and only 258 (49.3%) described upgrade instructions. Thirteen (2.5%) of the recalls due to software mentioned that a software upgrade would be available online. Nine (1.7%) mentioned that a software upgrade would *not* be available online. No Class I (high risk) recalls mentioned online updates; only five (1.0%) Class I recalls provided specific instructions for providers to upgrade software. Most Class I recalls were mitigated by manufacturer representatives upgrading software via either site visits or return shipping.

To further test the effectiveness of the FDA Safety Information and Adverse Event Reporting Program (MedWatch Form 3500) for reporting security and privacy problems, one co-author submitted a software vulnerability report for an automated external defibrillator on July 19, 2011.[18] As of January 19, 2012, the report had not yet been processed into MAUDE. In April 2012, MAUDE was found to contain the report for the event under report number MW5023578. The report processing took nine months. As the time from discovery of a conventional computer security vulnerability to the global exploitation of the flaw is often measured in hours, a nine month processing delay may not be an effective strategy for ensuring the security of software-based medical devices.

## Discussion

This study evaluated postmarket events in medical devices related to security and privacy using complementary databases compiling enforcement reports, recalls, and adverse events. Detailed review of enforcement reports revealed that recalls of devices with computers were common, though features such as wireless communication and storage of personal data were less common in those recalled devices. The FDA recall database did not yield any recalls related to patient security or privacy over a 9 year period of analysis. While the lack of any security or privacy concerns through these two mechanisms may be reassuring, it seems more likely that the current recall classification scheme does not adequately capture device malfunctions of this type. In addition, it is concerning that processing an adverse event report may take several months, given that a global exploitation of a security and privacy vulnerability may spread in a shorter period of time.

Our results also contrast with databases that track security and privacy problems for the Department of Veterans’ Affairs (VA). The Field Security Office in the Office of Information Security at the VA collects statistics on the prevalence of malicious software (malware) infections within its 156 medical centers. Between January 2009 and December 2011, the VA detected 142 separate instances of malware infections affecting 207 medical devices found in radiation oncology, radiology, clinical lab, GI lab, ophthalmology imaging, cardiology imaging, pharmacy, sleep lab, cardiac catheterization lab, pulmonary, dental, audiology, dictation, and neurology.[8] A common outcome was the unavailability of care because of computer outages. In one extreme instance, a computer virus infection in a catheterization lab required transport of patients to a different hospital. Common causes of infections include use of the Internet and USB flash memory drives from vendors who are paradoxically updating software on medical devices. In one instance, a factory-installed device arrived already infected with malware. All detected malware pertained to conventional computer viruses rather than malware customized for medical devices. The most prevalent malware converted the medical devices into becoming nodes of “botnet” criminal networks. Organized crime rents out botnets for others to distribute spam anonymously and for mounting targeted attacks on information infrastructure.

We believe that the inconsistency between databases is due to lack of a meaningful and convenient reporting mechanism, but we also believe that clinicians without expertise in computer security are unlikely to recognize the difference between a virus infection and a crashed or slow computer. Time pressure, lack of incentives, lack of federal safe harbor policies, and lack of clear actionable guidance likely further reduce the probability of incident reporting by clinicians and information technology staff.

Similarly, our review of the MAUDE database of adverse event reports did not identify any events related to privacy or security, despite inclusion of nearly 1000 possible product problems to facilitate targeted searching. Again, the negative findings here may be viewed in two ways. The absence of a glaring safety signal provides some reassurance that, for example, unauthorized access to patient information does not appear to be rampant. However, our manual review of the entire list of search product problems – from “abnormal” to “Y2K related problem”[14] – found only a handful of terms with a prima facie connection to security or privacy. This again suggests that the classification of postmarket events may not be well-positioned to prospectively collect security or privacy related problems. The detailed, verbatim review of the actual information provided in those adverse reports which mapped to security or privacy terms (**TABLE 2**) raises suspicions that current surveillance mechanisms may be insufficiently tailored to these specific problems.

This same concern is demonstrated in part by our findings related to software recalls. Most of these recalls indicated that a software update would be issued to correct the problem in question, but the mechanism of update itself remained unclear. These mechanisms might include web/internet based solutions, direct interventions by field engineers, or other interventions, each of which might introduce security risks. Our review of adverse events, however, suggests that even if an event were to occur – such as failure to update properly or deliberate interference with a software update – the current classification of “product problems” might not categorize these events clearly.

Our study reinforces findings of a prior evaluation of adverse events related to health information technology.[19] This much broader search strategy, also using MAUDE, found that only 0.1% of nearly 900,000 reports over a 2-year period were related to health information technology. These problems included a mix of software malfunctions, system configuration, and human errors. As with our report, these investigators suggested that the relatively low rate of findings may reflect known shortcomings of MAUDE, variability in reporting and the difficulty in even recognizing device malfunctions that are “unusual” or outside of traditional notions of device performance. Similarly, they identified a need for better design of prospective systems for capturing adverse events specific to the growing complexity of medical devices. Our contributions differ in two respects. First, our analysis is based on data from MAUDE as well as the FDA’s Enforcement Reports and Medical & Radiation Emitting Device Recalls. Second, our findings concern the issue of revising the current approach to postmarket surveillance to adequately identify problems related to the security and privacy of medical devices.

Our study has important limitations. As noted, our search strategy may not have been sufficient to identify reports or events related to privacy or security, although our manual review of search terms and reports was intentionally broad. All three databases focus on postmarket events that themselves required several links in a complex chain to become publicly known. Most importantly, device problems related to privacy and security must manifest clinically to become reportable, and by their very nature these issues may be difficult to detect. However, this strengthens our suggestion that better prospective mechanisms are needed to track device performance in this area.

The rapid proliferation of medical devices, and their growing sophistication, presents Internet-age challenges for multiple stakeholders. Without an understanding of security and privacy, it will be difficult for patients and clinicians to establish confidence in device safety and effectiveness. While this study provides some comfort in the lack of observed security or privacy breaches, the related adverse events or device malfunctions are not served well by the current approach to postmarket surveillance. This conclusion challenges regulators and manufacturers to carefully weigh the premarket evaluation of security and privacy elements of their devices and systems, and to design postmarket systems that enable effective collection of cybersecurity threat indicators for medical devices. While intentional interference may be much less likely to manifest clinically than other types of traditional malfunctions, it is clear that no effective system exists to detect signals of security or privacy problems. This conclusion is confirmed by the sharp contrast of security and privacy problems tabulated by the VA and the security and privacy problems tabulated with FDA databases. To detect a security or privacy problem that could harm patients, a more effective information sharing system for medical device cybersecurity should be established.
